# Establishment and application of real-time quantitative PCR for diagnosing invasive Aspergillosis via the blood in hematological patients: targeting a specific sequence of *Aspergillus* 28S-ITS2

**DOI:** 10.1186/1471-2334-13-255

**Published:** 2013-06-01

**Authors:** Yan Li, Li Gao, Yi Ding, Yuanyuan Xu, Minhang Zhou, Wenrong Huang, Yu Jing, Honghua Li, Lili Wang, Li Yu

**Affiliations:** 1 Department of Hematology and BMT center, Chinese PLA General Hospital, 28 Fuxing Road, Beijing 100853, China

**Keywords:** Hematological malignancies, Invasive aspergillosis, Real-time quantitative polymerase chain reaction, 28S-ITS2

## Abstract

**Background:**

Invasive aspergillosis (IA) is an important cause of morbidity and mortality in immunocompromised individuals. This study was conducted to identify a desirable target DNA sequence for the diagnosis of aspergillosis using real-time quantitative polymerase chain reaction (qPCR).

**Methods:**

Genomic DNA was extracted from *Aspergillus*, *Candida*, and bacteria species, and qPCR was applied to validate a partial ribosomal DNA 28S-ITS2 sequence. Ethylenediaminetetraacetic acid-anticoagulated blood samples were collected from 72 febrile hematological patients, while total DNA was isolated from plasma and whole blood for the *Aspergillus* qPCR. The results were analyzed using a receiver operating characteristic curve. All cases were evaluated using the revised European Organization for Research and Treatment of Cancer/Invasive Fungal Infections Cooperative Group and the National Institute of Allergy and Infectious Diseases Mycoses Study Group (EORTC/MSG) diagnostic criteria.

**Results:**

Use of qPCR yielded positive results for 15 *Aspergillus* species but negative results for *Candida* species, bacterial strains, and human DNA. The limit of detection was one copy per microliter of DNA. Analytical sensitivity and specificity were six copies of DNA and 100%, respectively. The standard curve showed that qPCR was reliable for *Aspergillus* detection and that significantly more DNA copies were obtained from whole blood than from plasma (*P* < 0.001). At a cut-off value ≥ 25 copies/μL, the diagnostic sensitivity and specificity for IA using 28S-ITS2 qPCR were 90.9% and 73.4%, respectively.

**Conclusions:**

The use of qPCR with whole blood to detect and verify the 28S-ITS2 sequence is a specific and useful way to diagnose IA.

## Background

Invasive aspergillosis (IA) is an important cause of morbidity and mortality in immunocompromised individuals. Patients at the highest risk are those with prolonged periods of neutropenia due to chemotherapy for acute leukemia or hematopoietic stem cell transplantation (HSCT) for hematological malignancies or solid tumors [1,2]. Conventional diagnostic tests such as blood cultures are not useful or practical for the diagnosis of *Aspergillus* spp. fungemia [3]. Further, non-culture–based techniques used in the past lack the sensitivity and specificity needed for the testing of immunocompromised patients [4-7]. Additionally, established IA is difficult to treat and has a death rate of 80–90%, which has driven researchers to develop more reliable and effective methods for its timely diagnosis [1,2,8,9]. Polymerase chain reaction (PCR) assays (conventional, nested, and real-time) have recently been developed for diagnosing fungal infections, especially quantitative real-time PCR (qPCR), which shows better potential for clinical application [1,10-14]. A range of gene region targets (cytochrome p450; heat shock proteins; the 18S, 5.8S, and 28S domains; and the internal transcribed space [ITS]) have been used in studies employing qPCR methods. Over the past few years, the ribosomal DNA gene region, composed of the *18S* (1800 bp), *5.8S* (159 bp), and *28S* (3396 bp) genes, has been shown to be a promising target in which the three above-mentioned genes are separated by the *ITS1* (361 bp) and *ITS2* (231 bp) genes. Until now, numerous studies have used target sequences from this region to detect *Aspergillus* DNA using PCR methods [10,15-18]. However, a consensus is not available with regard to the specific target sequence, specimen type, extraction method, or PCR format and platform. Hence, the objectives of our study were to search for a new potential target sequence for *Aspergillus*, assess the qPCR value, and determine the qPCR cut-off index for the presumptive clinical diagnosis of IA.

## Methods

### Preparation of fungal and bacterial cultures

All the strains were isolated from clinical patients. The tested strains consisted of: *Aspergillus* (15 strains), including *A. fumigatus* (four strains), *A. niger* (five strains), *A. flavus* (four strains), *A. nidulans* (one strain), and *A. terreus* (one strain); *Candida* (24 strains), including *C. albicans* (12 strains), *C. tropicalis* (four strains), *C. parapsilosis* (three strains), *C. pseudotropicalis* (one strain), *C. stellatoidea* (one strain), *C. krusei* (one strain), and *C. lipolytica* (one strain); *Monilia guilliermondii* (one strain); and assorted bacteria (10 strains) including *Staphylococcus aureus*, *Streptococcus pneumoniae*, *Neisseria meningitidis*, *Shigella*, *Haemophilus parainfluenzae*, *Escherichia coli*, *Klebsiella pneumoniae* (two strains)*,* and *Bacterium aeruginosum* (two strains).

Czapek’s agar and Sabouraud’s agar (Beijing AoBoXing Bio-Tech Co., Ltd., China) were inoculated with conidiospores from *Aspergillus* mycelia and *Candida*, respectively. Both were incubated at 30°C for 18–72 h until a mycelial mat formed. Blood agar, chocolate agar, and Mueller-Hinton agar plates (Beijing Laboratory Biology Technology of China, Beijing, China) were inoculated with different bacterial species and incubated at 37°C for 12–16 h. A proportion of the mycelial mat weighing approximately 10–20 mg was harvested with sterile forceps and placed into a sterile 1.5-mL Eppendorf tube with 600 μL of phosphate-buffered saline (PBS).

### Patient group

Seventy-two patients with hematological malignancies suffering from fever, four patients at normal temperature, and 10 healthy volunteers who visited our Hematology Department and Bone Transplantation Center between September 2010 and April 2011 were enrolled in the study. The study protocol was approved by the author’s institutional ethics committee, namely the Ethics Committee of General Hospital of Chinese Peoples’ Liberation Army, and was conducted in accordance with the Declaration of Helsinki. Written informed consent was obtained from all participants prior to specimen collection. Five-milliliter ethylenediaminetetraacetic acid (EDTA)-anticoagulated blood samples were collected when the patients were febrile [19], which was defined as an axillary temperature > 38.3°C on a single occasion or temperature > 38°C for >1 h (according to the National Comprehensive Cancer Network). An aliquot of 200 μL of plasma was centrifuged from the whole blood and total DNA was extracted from the plasma and the remnant whole blood. We then prospectively viewed the charts, electronic medical records, and laboratory records of these patients for the prior 60 days. IA was diagnosed on the basis of the revised European Organization for Research and Treatment of Cancer/Invasive Fungal Infections Cooperative Group and the National Institute of Allergy and Infectious Diseases Mycoses Study Group (EORTC/MSG) criteria [20]. Using these definitions, we classified the study population into four probability levels: proven IA, probable IA, possible IA, and no IA.

### DNA extraction

#### (i) From fungal specimens

The mycelial mats harvested in PBS were centrifuged for 1 min at 9,000 × *g*, and DNA was then extracted using a Biospin Fungus Genomic DNA Extraction Kit in accordance with the manufacturer’s instructions (BioFlux, China). In this process, a sample is first lysed in LE buffer, and then DNA in the sample is liberated. After DA buffer is added and the mixture is centrifuged at 14,000 × *g* for three minutes, the impurity is discarded. Released DNA is bound exclusively and specifically to the Biospin membrane in the presence of E Bingding Buffer under appropriate salt iron and pH conditions. Denatured proteins and other contaminants are removed by several washing procedures. The DNA is then eluted from the membrane using 30 μL of elution buffer.

#### (ii) From bacterial specimens

The bacterial harvest was centrifuged for one min at 12,000 × *g*, while genomic DNA was extracted using a Bacterial DNAout Kit according to the manufacturer’s instructions (Andy Bio, USA) for gram-positive and -negative bacteria.

#### (iii) From clinical specimens

Total DNA was simultaneously extracted from whole blood and 200 μL of supernatant plasma that had been centrifuged from the whole blood. Red blood cell lysis solution (Solarbio, China) was used to remove the red blood cells from the whole blood and obtain a white blood cell sediment. LE buffer was added to the white blood cells or 200 μL of plasma, and the following DNA extraction steps were undertaken in accordance with the manufacturer’s instructions of the Biospin Kit used for the fungal specimens. A 30-μL elution volume of DNA was ultimately obtained [19]. Human DNA was extracted using a DNA Purification Kit (Promega, USA) according to the relevant protocols.

As controls, an array of known positive samples from *A. fumigatus* and known negative samples from *C. albicans* was included in every set of DNA extractions. A maximum of 12 samples, including one positive and one negative control, was processed at any given time. The DNA concentration was determined from the absorbance at 260 nm measured using a UV spectrometer (Amersham Biosciences Co., USA).

### Primer and probe design

**Figure 1:**
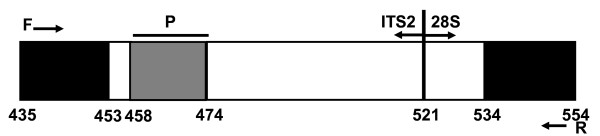
**The target sequence, primer, and probe.** The 1,154-bp sequence of ribosomal DNA includes an *18S* ribosomal RNA gene, a partial sequence (<0–12 bp), internal transcribed spacer 1 (ITS-1, 13–195 bp), the *5.8S* ribosomal RNA gene (196–352 bp), internal transcribed spacer 2 (ITS-2, 353–521 bp), 28S ribosomal RNA gene, and a partial sequence (522–>1,154 bp). The target sequence (435–554) includes ITS2 and 28S as well as the partial sequence 28S-ITS2.

Ribosomal subunit gene sequences of *Aspergillus* were accessed in NCBI GenBank (accession no. EF634384.1) and aligned using BLAST. Potential primer-binding sites were selected from the sequences conserved among *Aspergillus* (EF634384.1, 353–953 bp) but not among the human genome and other clinical relevant fungal genera except for *Penicillium* and *Paecilomyces* species. Primers were designed with the aid of Primer Express 3.0 software (Applied Biosystems,USA). The following primers targeting the 28S-ITS2 region of the ribosomal subunit gene, which was not examined in earlier studies, were used: forward primer, 5′-GTCCGGTCCTCGAGCGTAT-3′ (435–453); and reverse primer, 5′-GTTCAGCGGGTATCCCTACCT-3′ (534–554). They are approximately positioned on the *A. fumigatus* ribosomal 28S-ITS2 gene: GTCCGGTCCTCGAGCGTATGGGGCTTTGTCACCTGCTCTGTAGGCCCGGCCGGCGCCAGCCGACACCCAACTTTATTTTTCTAAGGTTGACCTCGGATCAGGTAGGGATACCCGCTGAAC (EF634384.1, 435–554 bp; ITS2, 435–521 bp; 28S, 522–554 bp). The hydrolysis probe sequence was fluorescence labeled with 6-carboxyfluorescein at the 5' end as the reporter dye and None Fluorescence Quencher modified by Minor Groove Binder at the 3' end as the quencher 5′-FAM-CTTTGTCACCTGCTCTG-NFQ-MGB-3′ (458–474) (Figure 1).

### Real-time qPCR

To minimize contamination, the PCR assays were set up in a class 2 laminar flow cabinet in a separate room from the DNA extractions. The qPCR procedure was performed using 40-μL reaction mixes on Mx3000P (Stratagene, USA). Each reaction contained 20 μL of 2× QuantiTect Probe PCR Master Mix Buffer (Qiagen, Germany), 1 μL of each primer, 0.5 μL of the probe (American Biosystems, USA), 6 μL of template DNA, and 11.5 μL of RNase-Free Water (Qiagen). The conditions for the qPCR were as follows: 50°C for 2 min to digest the PCR products including dUMP using uracil-*N*-glycosylase; 95°C for 10 min to activate a hot start DNA polymerase; and 40 cycles of denaturing at 94°C for 15 sec and annealing and extension at 59°C for 1 min. Each sample was processed in a set of two counterparts. When the difference in the quantification cycle (Cq) value of the two counterparts was >1, the sample processing was repeated in another qPCR set until the Cq difference was <1 cycle to ensure concordance and accuracy. Two positive controls containing copies of *A. fumigatus*, two negative controls containing copies of *C. albicans,* and two blank controls containing water were also included with each run to monitor inter-assay consistency. All DNA extractions and qPCR procedures were performed with the operator blinded to the strains’ species and the patients’ clinical features. The results were shown by initial quantity (copies/μL).

### Standard preparation

The amplified DNA products of *A. fumigatus* were run on a 1.8% agarose gel and extracted using a QIAquick Gel Extraction Kit according to the manufacturer’s instructions (Qiagen). The purified target DNA was ligated into a pGEM-TEasy Vector System I (A1360; Promega), while the recombinant plasmid was transformed into *Escherichia coli*-competent DH5α cells (Beijing BioMedTech Co., Ltd., China) and extracted using a Wizard Plus SV Miniprep DNA Purification Kit (Promega) according to the manufacturer’s instructions. The recombinant plasmid DNA was run on an agarose gel with positive and negative controls and then amplified and identified. The predicted product size was 120 bp. The transformation was also confirmed by sequencing the target fragment using liquid cultures of *E. coli*. After identification, the recombinant plasmid DNA was diluted 10-fold from 1 × 10^9^ copies/μL to 1 × 10^3^ copies/μL with RNase-free water (Qiagen) for a series of standard qPCR preparations used to generate the standard curve. Each sample was processed in a set of triplicate counterparts.

### Calculation of *Aspergillus* qPCR cut-off value

Both cases of proven and probable IA were assumed to be true. The other patients and the 10 healthy volunteers were considered non-IA in the analysis. The initial qPCR copies for every case were included in the calculation. The cut-off value of the qPCR was determined using receiver operating characteristic (ROC) analysis. Sensitivity and specificity were calculated using IA cases as the reference standard.

### Statistical analysis

Continuous variables were compared using a nonparametric test (Mann–Whitney *U* test). Two-tailed *P* values < 0.05 were considered statistically significant. An appropriate qPCR cut-off value was estimated using ROC analysis. Statistical analyses were performed using SPSS software version 19.0 (IBM, USA).

## Results

### Real-time qPCR development

The qPCR protocol was optimized with respect to primer and probe concentrations, DNA template quantities, buffer composition, number of cycles, and cycle parameters (data not shown). The qPCR yielded positive results for all 15 tested isolates of *Aspergillus*: the mean Cq value was 21.66 (95% confidence interval [CI], 20.05–23.27), while the mean initial number of DNA copies was 2.47 × 10^6^/μL (95% CI, 1.69 × 10^5^–5.11 × 10^6^/μL). None of the 24 *Candida* samples or the 10 bacterial and human DNA samples tested was amplified. Thus, the analytical specificity of this method was 100%. The use of serial 10-fold dilutions of *A. fumigatus* DNA in water showed that the qPCR limit of detection was one copy per microliter of DNA with the Cq value around 37, while the analytical sensitivity was six copies of DNA.

The recombinant plasmid DNA was identified by running on an agarose gel and sequencing. It was then diluted for a series of standard preparations and subjected to qPCR. The standard curve was Cq = −3.332 × log (initial quantity, copies/μL) + 40.75 (RSq = 0.991; Eff = 99.6%).

### Characteristics of the study population

A total of 72 patients (30 women, 42 men) were included in the study (Table 1). The median age was 42.5 years (range, 3–76 years). Forty (55.6%) patients had acute leukemia, while the other 25 (34.7%) had undergone HSCT. According to the EORTC/MSG criteria [20], IA was diagnosed in 41 patients (four patients with proven IA confirmed via lung biopsy, 18 with probable IA, and 19 with possible IA), and the 28S-ITS2 qPCR results (median, range) for proven, probable, and possible IA were 123.47 (25.96–266.95), 43.65 (0–549.5), and 16.15 (0–98.76) copies/μL (Table 2). No amplification was seen in the controls (four patients with normal temperature and the 10 healthy volunteers). Febrile neutropenia was the most common in-host factor, while the halo sign (78%) was the most common clinical criterion. No patients were tested using the galactomannan (GM) test, and the mycological criteria were judged mainly according to sputum microscopic evaluation or culture results (Table 2).

**Table 1:** Clinical features of febrile patients with hematological malignancies

**Demographic/characteristic**	**No. patients (%) (n = 72)**
Age (years)	
Median	42.5
Range	3–76
Gender	
Male	42 (58.3)
Female	30 (41.7)
Hematological malignancy	
AML	30 (41.7)
ALL	10 (13.9)
CML	3 (4.2)
CMML	1 (1.4)
CLL	2 (2.8)
NHL	14 (19.4)
MDS	7 (9.7)
MM	5 (6.9)
HSCT	25 (34.7)
Allo-HSCT	21 (29.2)
Auto-HSCT	4 (5.6)
IA	41 (56.9)
Proven	4 (5.6)
Probable	18 (25)
Possible	19 (26.4)
Sites	
Pulmonary	40 (55.6)
Systemic	1 (1.4)

AML, acute myeloid leukemia; ALL, acute lymphoblastic leukemia; CML, chronic myelogenous leukemia; CMML, chronic myelomonocytic leukemia; CLL, chronic lymphocytic leukemia; NHL, non-Hodgkin’s lymphoma; MDS, myelodysplastic syndromes; MM, multiple myeloma; HSCT, hematopoietic stem cell transplantation; IA, invasive aspergillosis.

**Table 2:** Diagnostic criteria and 28S-ITS2 qPCR results for invasive aspergillosis

**Diagnostic criteria**	**IA category**			**Total (%) (n=41)**
	**Proven (%) (n=4)**	**Probable (%) (n=18)**	**Possible (%) (n=19)**	
**Host factors**				
Neutropenia	4 (100)	6 (33.3)	3 (15.8)	13 (31.7)
T > 38°C with				
Prolonged neutropenia	4 (100)	11 (61.1)	6 (31.6)	21 (51.2)
Immunosuppressant	1 (25)	9 (50)	9 (47.4)	19 (46.3)
Previous IFI	0 (0)	4 (22.2)	3 (15.8)	7 (17.1)
With AIDS	0 (0)	0 (0)	0 (0)	0 (0)
GVHD	0 (0)	3 (16.7)	2 (10.5)	5 (12.2)
Corticosteroids	3 (75)	4 (22.2)	7 (36.8)	14 (34.1)
**Clinical criteria**				
Halo sign	3 (75)	17 (94.4)	12 (63.1)	32 (78)
Air-crescent sign	2 (50)	1 (5.6)	2 (10.5)	5 (12.2)
Cavity	1 (25)	3 (16.7)	0 (0)	4 (9.8)
Symptoms of LRI	3 (75)	8 (44.4)	13 (68.4)	24 (58.5)
Permanent fever	2 (50)	7 (38.9)	13 (68.4)	22 (53.7)
**Mycological criteria**				
Positive sputum microscopy	0(0)	6 (33.3)	0 (0)	6 (14.6)
Positive sputum culture	1 (25)	5 (27.8)	0 (0)	6 (14.6)
G test positive	1 (25)	8 (44.4)	1 (5.3)	10 (24.4)
No bacterial positive	0 (0)	6 (33.3)	3 (15.8)	9 (22)
**Histology**				
Biopsy specimen of the lung	4 (100)	0 (0)	0 (0)	4 (9.8)
**qPCR results (copies/μL) (median, range)**	123.47	43.65	16.15	28.12
	(25.96–266.95)	(0–549.5)	(0–98.76)	(0–549.5)

qPCR, real-time qualitative polymerase chain reaction; IA, invasive aspergillosis; IFI, invasive fungal infections; AIDS, acquired immune deficiency syndrome; GVHD, graft versus host disease; LRI, lower respiratory infections.

### Calculating the cut-off value for the *Aspergillus* 28S-ITS2 qPCR

Whole blood and plasma samples were simultaneously collected from the patients for *Aspergillus* DNA isolation and purification, and the qPCR assays were then performed using the hydrolysis probe method. Significantly greater numbers of DNA copies were amplified by qPCR from whole blood than from plasma (28.36 vs. 7.28, *P* < 0.001), and DNA was not amplified from most of the plasma samples. Therefore, the whole blood qPCR results were used in the subsequent analysis. A significantly higher median number of initial quantity was amplified from patients with proven and probable IA (57.87 copies/μL; range, 0–549.5) than patients without IA (*P* < 0.001).

**Figure 2:**
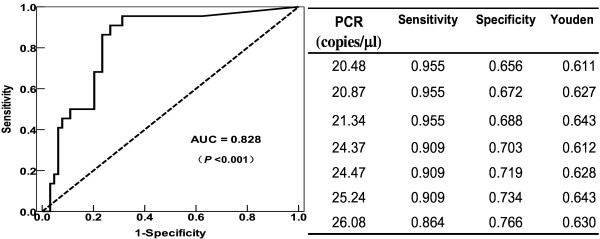
**The receiver operating characteristic (ROC) analysis.** The ROC curve and table of statistics for the cut-off value for the group of patients with proven and probable invasive aspergillosis were used as standards.

Using proven and probable IA cases as the reference standard, the area under the ROC curve (AUC) was 0.828 (95% CI, 0.733–0.923; *P* < 0.001). The optimal infection point was located between a qPCR index of 21.34 (sensitivity, 95.5%; specificity, 68.8%; Youden index, 0.643) and 25.24 (sensitivity, 90.9%; specificity, 73.4%; Youden index, 0.643) (Figure 2). According to the ROC results and considering the clinical situation in which the rate of missed diagnosis is very high compared to the misdiagnosis rate, and if the diagnosis is missed in patients with IA, the prognosis is very poor, so we chose a threshold with a high sensitivity and a suboptimal specificity. In this study, qPCR was considered positive when there were ≥25 copies/μL (Table 3). Based on this cut-off value, the diagnostic sensitivity and specificity for IA by 28S-ITS2 real-time PCR were 90.9% and 73.4%, respectively.

**Table 3:** The diagnostic results of qPCR and EORTC/MSG criteria

**Reference standard according to IA category**	**qPCR (≥25copies/μL)**		**Total**
	**Positive**	**Negative**	
Positive			22
Proven	4	0	
Probable	16	2	
Negative			64
Possible	7	12	
No IA	10	35	
Total	37	49	86

qPCR, real-time qualitative polymerase chain reaction; EORTC/MSG, European Organization for Research and Treatment of Cancer/Invasive Fungal Infections Cooperative Group and the National Institute of Allergy and Infectious Diseases Mycoses Study Group; IA, invasive aspergillosis.

## Discussion

The results of this study showed that the target region of 28S-ITS2 is specific and applicable to the genetic diagnosis of *Aspergillus* and that the whole blood qPCR assay may be a very useful and reliable method for the early diagnosis of IA in patients with hematological malignancies.

Over the past decade, many researchers have explored and evaluated PCR assays for the early diagnosis of IA [10,21-24], while the European *Aspergillus* PCR Initiative (EAPCRI) has performed a lot of meaningful work around PCR standardization [13,19,25]. However, PCR is still not included in the EORTC/MSG criteria [16]. Meanwhile, there is a major consensus in favor of qPCR technology due to its sensitivity and speed [13,16,24,26-28], especially after release of the Minimum Information for Publication of Quantitative Real-Time PCR Experiments (MIQE) guidelines, which has encouraged better experimental practice for more reliable and unequivocal interpretation of qPCR results [14,29]. A critical issue of qPCR is the choice of the target DNA, which is highly debated since it is directly associated with assay sensitivity and specificity. To date, several genes have been used to assess qPCR assay results in the diagnosis of IA [10,11,15], and the ribosomal DNA gene region has been used more and more frequently and shows the greatest potential [16-18,25,29,30]. Ribosomal DNA comprises transcriptional domains (5S, 5.8S, 18S, and 28S), which evolve relatively slowly and have higher conservation rates, and non-transcriptional domains (ITS1 and ITS2), which are more variable [31]. A multicenter study comparing the use of the 28S and 18S primer sets targeting the D1–D2 region of the large ribosomal subunit gene revealed that the former was more specific, probably because when the 18S region is targeted, a portion of the human rDNA gene needs to be amplified, especially in the absence of *A. fumigatus* DNA [18,32].

Overall, use of the 28S primer set is more suitable than the 18S primer set for the diagnosis of *Aspergillus*. The primers in our study were derived from the 28S-ITS2 region in the GenBank database and targeted both the conservative and variable regions as described before, while the probe was modified by minor groove binder, which further confirmed its specificity. The target sequence was aligned using BLAST and tested for analytic sensitivity and specificity using 15 *Aspergillus* species, 24 *Candida* species*,* 10 bacterial species, and serial 10-fold dilutions of *A. fumigatus* DNA, with the results of 6 copies and 100%, respectively, indicating the applicability of the *Aspergillus* qPCR assay.

With regard to the best blood fraction to test, specimen choice had critical implications in the extraction methodology and PCR results. Historically, the pathogenesis of IA has been poorly understood, and knowledge regarding the source of *Aspergillus* nucleic acids in the blood is limited with no consensus having been achieved to date. Testing serum specimens using PCR is dependent on the detection of free circulating *Aspergillus* DNA, while the use of EDTA-anticoagulated whole blood samples allows for the detection of conidia, hyphal fragments, and freely circulating DNA [12,18]. In our study, plasma and whole blood were compared for *Aspergillus* DNA extraction, and the results showed greater numbers of initial DNA copies in whole blood than in plasma. Thus, whole blood was the better specimen for *Aspergillus* DNA extraction, a finding that was consistent with those of earlier reports and the EAPCRI recommendation [12,13,33].

To initially assess the clinical applicability of this *Aspergillus* qPCR, 86 blood samples from 86 individuals were tested: 72 of these individuals were febrile and had hematological malignancies, while 41 were diagnosed with IA according to clinical criteria. The qPCR results and clinical data were analyzed using ROC [27]. The main obstacle we encountered was that the number of proven cases of aspergillosis was too low to be used as the sole criterion for diagnosis since the blood cultures were almost always negative for *Aspergillus* species. The probability of confirming the diagnosis histopathologically was also low because patient status was not suitable for invasive diagnostic procedures. According to the qPCR results, the proven and probable cases were defined as being within the reference standard category on the basis of the EORTC/MSG criteria, the only accepted tool, although it has some limitations [20,34].

The choice of an optimal cut-off value depends on the test purpose. An ROC analysis was conducted that revealed a good AUC value (0.828). In clinical practice, there are few effective clinical approaches for the diagnosis of IA; once a diagnosis is missed, patient prognosis is very poor. Hence, we chose a qPCR index that yielded an adequately high sensitivity, an acceptable false-positive rate (1-specificity), and a better Youden index, which reflects integrated diagnostic ability. In the ROC analysis, a cut-off value of 25.24 seemed to yield the optimal trade-off (Figure 2). The qPCR cut-off value of ≥25 copies/μL was tentatively established as the most suitable for obtaining a high yield in clinical screening for IA, while the Cq value was 36, consistent with that of the EAPCRI report (mean, 35.3; upper limit, 38.8 cycles) [13]. According to this threshold value, the diagnostic sensitivity and specificity were 90.9% and 73.4%, respectively, while meta-analysis showed that these values would be 88% (95% CI, 75–94%) and 75% (95% CI, 63–84%) if only a single positive sample was required [35]. With this threshold, the specificity (73.4%) seemed suboptimal, and 41% (7/17) of the patients with false-positive results were diagnosed as possible IA and 59% (10/17) were diagnosed as no IA, which accounted for the possible IA and no IA rates of 37% (7/19) and 22% (10/45), respectively (Table 3). We analyzed these patients and speculated that their degraded diagnoses were mainly due to the limitations of the EORTC/MSG criteria [34] and the fact that high sensitivity can also cause false-positive results due to low-level contamination [36]. Due to the heterogeneity of the different studies, there is no way to compare them. Of course, increasing the size of the study population, especially the number of proven cases, may result in a better cut-off index and a more suitable guide for the presumptive diagnosis of IA in future studies.

Another limitation of this study was that we did not collect serial specimens for qPCR since we immediately administered empirical antifungal treatment to patients with neutropenic fever, which was thought to affect the diagnostic detection [37]. Otherwise, no association was found between *Aspergillus* DNA detection and treatment efficacy or patient outcome [38-41], the probable reason for this being the lower DNA loads in the blood, which made reliable quantification difficult due to the random dispersion of the few available DNA copies [39]. The current use of *Aspergillus* qPCR as a diagnostic screening tool is recommended instead of evaluation for therapy, and a single PCR-negative result is sufficient to exclude the diagnosis of IA because of this assay’s high sensitivity [10,16,42]. The GM test was not performed simultaneously with the one-time qPCR for each patient in this study since *Aspergillus* GM and DNA release do not occur in a parallel fashion due to IA pathogenesis and host factor changes that occur over time [43-45]. Additionally, several studies have indicated that qPCR was comparable or better to GM for IA diagnosis and that it is more scientific and appropriate to combine GM and qPCR for IA diagnosis [42,45-47].

## Conclusions

Here we found a specific sequence of *Aspergillus* 28S-ITS2 for qPCR that offers good sensitivity and specificity. This assay was initially assessed in patients with hematological malignancies, and a tentative cut-off value for the diagnosis of IA was obtained. Future clinical studies are necessary, especially diagnostic randomized controlled trials for this assay using the novel superior DNA extraction method and ultraclean production according to the MIQE guidelines [14,36,43]. We expect that this qPCR assay will be useful for early diagnosing to enable the initiation of preemptive IA treatment.

## Abbreviations

28S-ITS2: Partial sequence of the ribosomal DNA including the 28S domain and internal transcribed space 2 domain; IA: Invasive aspergillosis; qPCR: Real-time quantitative PCR; EORTC/MSG: European Organization for Research and Treatment of Cancer/Invasive Fungal Infections Cooperative Group and the National Institute of Allergy and Infectious Diseases Mycoses Study Group; Cq: Quantification cycle; ROC: Receiver operating characteristic; GM: Galactomannan; AUC: Area under the ROC curve.

## Competing interests

The authors declare that they have no competing interests.

## Authors’ contributions

YL, LG, and LY designed this study. YD and YX performed the PCR assays and data collection. YL and LG performed the data analysis, calculated the statistics, and drafted the article, while LW and LY critically revised the article. WH, YJ, and HL provided valuable advice, supported the clinical protocol, and edited the article. Study funding was secured by LY. All of the authors have read and approved the final manuscript.

## Pre-publication history

The pre-publication history for this paper can be accessed here:

http://www.biomedcentral.com/1471-2334/13/255/prepub

## References

[B1] Morgan J Wannemuehler KA Marr KA Hadley S Kontoyiannis DP Walsh TJ Fridkin SK Pappas PG Warnock DW Incidence of invasive aspergillosis following hematopoietic stem cell and solid organ transplantation: interim results of a prospective multicenter surveillance program Med Mycol 2005 43 Suppl 1 S49 S58 16110792 10.1080/13693780400020113

[B2] Upton A Kirby KA Carpenter P Boeckh M Marr KA Invasive aspergillosis following hematopoietic cell transplantation: outcomes and prognostic factors associated with mortality Clin Infect Dis 2007 44 531 540 10.1086/510592 17243056

[B3] Meyer MH Letscher-Bru V Jaulhac B Waller J Candolfi E Comparison of mycosis IC/F and plus aerobic/F media for diagnosis of fungemia by the bactec 9240 system J Clin Microbiol 2004 42 773 777 10.1128/JCM.42.2.773-777.2004 14766852 PMC344513

[B4] Asano-Mori Y Kanda Y Oshima K Kako S Shinohara A Nakasone H Kaneko M Sato H Watanabe T Hosoya N Izutsu K Asai T Hangaishi A Motokura T Chiba S Kurokawa M False-positive Aspergillus galactomannan antigenaemia after haematopoietic stem cell transplantation J Antimicrob Chemother 2008 61 411 416 18055488 10.1093/jac/dkm463

[B5] Segal BH Walsh TJ Current approaches to diagnosis and treatment of invasive aspergillosis Am J Respir Crit Care Med 2006 173 707 717 10.1164/rccm.200505-727SO 16387806

[B6] Horger M Einsele H Schumacher U Wehrmann M Hebart H Lengerke C Vonthein R Claussen CD Pfannenberg C Invasive pulmonary aspergillosis: frequency and meaning of the "hypodense sign" on unenhanced CT Br J Radiol 2005 78 697 703 10.1259/bjr/49174919 16046420

[B7] Greene RE Schlamm HT Oestmann JW Stark P Durand C Lortholary O Wingard JR Herbrecht R Ribaud P Patterson TF Troke PF Denning DW Bennett JE de Pauw BE Rubin RH Imaging findings in acute invasive pulmonary aspergillosis: clinical significance of the halo sign Clin Infect Dis 2007 44 373 379 10.1086/509917 17205443

[B8] Robenshtok E Gafter-Gvili A Goldberg E Weinberger M Yeshurun M Leibovici L Paul M Antifungal prophylaxis in cancer patients after chemotherapy or hematopoietic stem-cell transplantation: systematic review and meta-analysis J Clin Oncol 2007 25 5471 5489 10.1200/JCO.2007.12.3851 17909198

[B9] Hot A Maunoury C Poiree S Lanternier F Viard JP Loulergue P Coignard H Bougnoux ME Suarez F Rubio MT Mahlaoui N Dupont B Lecuit M Faraggi M Lortholary O Diagnostic contribution of positron emission tomography with [18F] fluorodeoxyglucose for invasive fungal infections Clin Microbiol Infect 2011 17 409 417 10.1111/j.1469-0691.2010.03301.x 20636432

[B10] Schabereiter-Gurtner C Selitsch B Rotter ML Hirschl AM Willinger B Development of novel real-time PCR assays for detection and differentiation of eleven medically important aspergillus and candida species in clinical specimens J Clin Microbiol 2007 45 906 914 10.1128/JCM.01344-06 17251398 PMC1829149

[B11] White PL Barnes RA Aspergillus PCR-platforms, strengths and weaknesses Med Mycol 2006 44 191 198 10.1080/13693780600898003 30408903

[B12] Klingspor L Loeffler J Aspergillus PCR formidable challenges and progress Med Mycol 2009 47 Suppl 1 S241 S247 19253138 10.1080/13693780802616823

[B13] White PL Bretagne S Klingspor L Melchers WJ McCulloch E Schulz B Finnstrom N Mengoli C Barnes RA Donnelly JP Loeffler J Aspergillus PCR: One step closer to standardization J Clin Microbiol 2010 48 1231 1240 10.1128/JCM.01767-09 20147637 PMC2849576

[B14] Bustin SA Benes V Garson JA Hellemans J Huggett J Kubista M Mueller R Nolan T Pfaffl MW Shipley GL Vandesompele J Wittwer CT The MIQE guidelines: minimum information for publication of quantitative real-time PCR experiments Clin Chem 2009 55 611 622 10.1373/clinchem.2008.112797 19246619

[B15] Klingspor L Jalal S Molecular detection and identification of Candida and aspergillus spp. From clinical samples using real-time PCR Clin Microbiol Infect 2006 12 745 753 10.1111/j.1469-0691.2006.01498.x 16842569

[B16] White PL Linton CJ Perry MD Johnson EM Barnes RA The evolution and evaluation of a whole blood polymerase chain reaction assay for the detection of invasive aspergillosis in hematology patients in a routine clinical setting Clin Infect Dis 2006 42 479 486 10.1086/499949 16421791

[B17] Cesaro S Stenghele C Calore E Franchin E Cerbaro I Cusinato R Tridello G Manganelli R Carlli M Palu G Assessment of the lightcycler PCR assay for diagnosis of invasive aspergillosis in paediatric patients with onco-haematological diseases Mycoses 2008 51 497 504 10.1111/j.1439-0507.2008.01512.x 18331444

[B18] White PL Barton R Guiver M Linton CJ Wilson S Smith M Gomez BL Carr MJ Kimmitt PT Seaton S Rajakumar K Holyoake T Kibbler CC Johnson E Hobson RP Jones B Barnes RA A consensus on fungal polymerase chain reaction diagnosis?: a United Kingdom-Ireland evaluation of polymerase chain reaction methods for detection of systemic fungal infections J Mol Diagn 2006 8 376 384 10.2353/jmoldx.2006.050120 16825512 PMC1867606

[B19] White PL Perry MD Loeffler J Melchers W Klingspor L Bretagne S McCulloch E Cuenca-Estrella M Finnstrom N Donnelly JP Barnes RA European Aspergillus PCR Initiative Critical stages of extracting DNA from Aspergillus fumigatus in whole-blood specimens J Clin Microbiol 2010 48 3753 3755 10.1128/JCM.01466-10 20720026 PMC2953093

[B20] De Pauw B Walsh TJ Donnelly JP Stevens DA Edwards JE Calandra T Pappas PG Maertens J Lortholary O Kauffman CA Denning DW Patterson TF Maschmeyer G Bille J Dismukes WE Herbrecht R Hope WW Kibbler CC Kullberg BJ Marr KA Muñoz P Odds FC Perfect JR Restrepo A Ruhnke M Segal BH Sobel JD Sorrell TC Viscoli C Wingard JR Revised definitions of invasive fungal disease from the European Organization for Research and Treatment of Cancer/invasive fungal infections cooperative group and the national institute of allergy and infectious diseases Mycoses Study Group (EORTC/MSG) consensus group Clin Infect Dis 2008 46 1813 1821 10.1086/588660 18462102 PMC2671227

[B21] Asano-Mori Y Fungal infections after hematopoietic stem cell transplantation Int J Hematol 2010 91 576 587 10.1007/s12185-010-0574-0 20432074

[B22] Halliday C Hoile R Sorrell T James G Yadav S Shaw P Bleakley M Bradstock K Chen S Role of prospective screening of blood for invasive aspergillosis by polymerase chain reaction in febrile neutropenic recipients of haematopoietic stem cell transplants and patients with acute leukaemia Br J Haematol 2006 132 478 486 16412020 10.1111/j.1365-2141.2005.05887.x

[B23] Loffler J Hebart H Sepe S Schumcher U Klingebiel T Einsele H Detection of PCR-amplified fungal DNA by using a PCR-ELISA system Med Mycol 1998 36 275 279 10.1080/02681219880000441 10075496

[B24] Somogyvari F Horvath A Serly J Majoros H Vagvolgyi C Peto Z Detection of invasive fungal pathogens by real-time PCR and high-resolution melting analysis In Vivo 2012 26 979 983 23160681

[B25] White PL Mengoli C Bretagne S Cuenca-Estrella M Finnstrom N Klingspor L Melchers WJ McCulloch E Barnes RA Donnelly JP Loeffler J European Aspergillus PCR Initiative Evaluation of Aspergillus PCR protocols for testing serum specimens J Clin Microbiol 2011 49 3843 3848

[B26] Millon L Grenouillet F Legrand F Loewert S Bellanger AP Gbaguidi-Haore H Scherer E Henon T Rohrlich P Deconinck E Ribosomal and mitochondrial DNA target for real-time PCR diagnosis of invasive Aspergillosis J Clin Microbiol 2011 49 1058 1063 10.1128/JCM.01904-10 21227993 PMC3067699

[B27] Yoo JH Choi JH Choi SM Lee DG Shin WS Min WS Kim CC Application of nucleic acid sequence-based amplification for diagnosis of and monitoring the clinical course of invasive aspergillosis in patients with hematologic diseases Clin Infect Dis 2005 40 392 398 10.1086/427284 15668862

[B28] Bašková L Buchta V Laboratory diagnostics of invasive fungal infections: an overview with emphasis on molecular approach Folia Microbiol 2012 57 421 430 10.1007/s12223-012-0152-3 22566119

[B29] Johnson GL Bibby DF Wong S Agrawal SG Bustin SA MIQE-compliant real-time PCR assay for Aspergillus detection PLoS One 2012 7 e40022 10.1371/journal.pone.0040022 22808087 PMC3393739

[B30] Ogawa M Sugita S Watanabe K Shimizu N Mochizuki M Novel diagnosis of fungal endophthalmitis by broad-range real-time PCR detection of fungal 28S ribosomal DNA Graefes Arch Clin Exp Ophthalmol 2012 250 1877 1883 10.1007/s00417-012-2015-7 22527320

[B31] Einsele H Hebart H Roller G Loffler J Rothenhofer I Muller CA Bowden RA van Burik J Engelhard D Kanz L Schumacher U Detection and identification of fungal pathogens in blood by using molecular probes J Clin Microbiol 1997 35 1353 1360 9163443 10.1128/jcm.35.6.1353-1360.1997 PMC229748

[B32] Williamson EC Leeming JP Palmer HM Steward CG Warnock D Marks DI Millar MR Diagnosis of invasive aspergillosis in bone marrow transplant recipients by polymerase chain reaction Br J Haematol 2000 108 132 139 10.1046/j.1365-2141.2000.01795.x 10651736

[B33] Loeffler J Hebart H Brauchle U Schumacher U Einsele H Comparison between plasma and whole blood specimens for detection of Aspergillus DNA by PCR J Clin Microbiol 2000 38 3830 3833 11015412 10.1128/jcm.38.10.3830-3833.2000 PMC87485

[B34] Tsitsikas DA Morin A Araf S Murtagh B Johnson G Vinnicombe S Ellis S Suaris T Wilks M Doffman S Agrawal SG Impact of the revised (2008), EORTC/MSG definitions for invasive fungal disease on the rates of diagnosis of invasive aspergillosis Med Mycol 2012 50 538 542 10.3109/13693786.2011.630040 22074309

[B35] Mengoli C Cruciani M Barnes RA Loeffler J Donnelly JP Use of PCR for diagnosis of invasive aspergillosis: systematic review and meta-analysis Lancet Infect Dis 2009 9 89 96 10.1016/S1473-3099(09)70019-2 19179225

[B36] Springer J Schloßnagel H Heinz W Doedt T Soeller R Einsele H Loeffler J A novel extraction method combining plasma with whole blood fraction shows excellent sensitivity and reproducibility in patients at high risk for invasive aspergillosis J Clin Microbiol 2012 50 2585 2591 10.1128/JCM.00523-12 22593600 PMC3421527

[B37] McCulloch E Ramage G Rajendran R Lappin DF Jones B Warn P Shrief R Kirkpatrick WR Patterson TF Williams C Antifungal treatment affects the laboratory diagnosis of invasive aspergillosis J Clin Pathol 2012 65 83 86 10.1136/jcp.2011.090464 22049217

[B38] Bergeron A Porcher R Menotti J Poirot JL Chagnon K Vekhoff A Cornet M Isnard F Raffoux E Brethon B Lacroix C Touratier S Latgé JP Bouges-Michel C Tazi A Derouin F Ribaud P Sulahian A Prospective evaluation of clinical and biological markers to predict the outcome of invasive pulmonary Aspergillosis in hematological patients J Clin Microbiol 2012 50 823 830 10.1128/JCM.00750-11 22170907 PMC3295141

[B39] Bretagne S Primary diagnostic approaches of invasive Aspergillosis -molecular testing Medical Mycology Month 2011 49 S48 S53 10.3109/13693786.2010.508186 20718612

[B40] Xu MZ Antigen and molecular biology research on early diagnosis of invasive Aspergillosis in patient with hematologic malignancie 2009 http://d.wanfangdata.com.cn/Thesis_Y1483956.aspx

[B41] Cuenca-Estrella M Meije Y Diaz-Pedroche C Gomez-Lopez A Buitrago MJ Bernal-Martinez L Grande C Juan RS Lizasoain M Rodriguez-Tudela JL Aguado JM Value of serial quantification of fungal DNA by a real-time PCR-based technique for early diagnosis of invasive Aspergillosis in patients with febrile neutropenia J Clin Microbiol 2009 47 379 384 10.1128/JCM.01716-08 19109479 PMC2643681

[B42] Mennink-Kersten MA Donnelly JP Verweij PE Detection of circulating galactomannan for the diagnosis and management of invasive Aspergillosis Lancet Infect Dis 2004 4 349 357 10.1016/S1473-3099(04)01045-X 15172343

[B43] Morrissey CO Chen SC Sorrell TC Bradstock KF Szer J Halliday CL Gilroy NM Schwarer AP Slavin MA Design issues in a randomized controlled trial of a pre-emptive versus empiric antifungal strategy for invasive Aspergillosis in patients with high-risk hematologic malignancies Leuk Lymphoma 2011 52 179 193 10.3109/10428194.2010.542600 21281234

[B44] Balloy V Huerre M Latge JP Chignard M Differences in patterns of infection and inflammation for corticosteroid treatment and chemotherapy in experimental invasive pulmonary Aspergillosis Infect Immun 2005 73 494 503 10.1128/IAI.73.1.494-503.2005 15618189 PMC538925

[B45] Aquino VR Nagel F Andreolla HF de-Paris F Xavier MO Goldani LZ Denning DW Pasqualotto AC The performance of real-time PCR, Galactomannan, and fungal culture in the diagnosis of invasive Aspergillosis in ventilated patients with chronic obstructive pulmonary disease (COPD) Mycopathologia 2012 174 163 169 10.1007/s11046-012-9531-1 22382738

[B46] Ostrosky-Zeichner L Invasive mycoses: diagnostic challenges Am J Med 2012 125 Suppl S14 S24 22196205 10.1016/j.amjmed.2011.10.008

[B47] Springer J Morton CO Perry M Heinz WJ Paholcsek M Alzheimer M Rogers TR Barnes RA Einsele H Loeffler J White PL Comparison of serum and whole blood specimens for the detection of Aspergillus DNA in high-risk haematological patients: a multicenter evaluation J Clin Microbiol 2013 Epub ahead of print 10.1128/JCM.03322-12 PMC3647891 23426930

